# How Can Journals Respond to Threats of Libel Litigation?

**DOI:** 10.1371/journal.pmed.1001615

**Published:** 2014-03-25

**Authors:** Nav Persaud, Thom Ringer, Trudo Lemmens

**Affiliations:** 1 Keenan Research Centre in the Li Ka Shing Knowledge Institute, St Michael's Hospital, Toronto, Ontario, Canada; 2 Department of Family and Community Medicine, University of Toronto, Toronto, Ontario, Canada; 3 Department of Family and Community Medicine, St Michael's Hospital, Toronto, Ontario, Canada; 4 Faculty of Law, University of Toronto, Toronto, Ontario, Canada; 5 Joint Centre for Bioethics, University of Toronto, Toronto, Ontario, Canada; Journal of the RSM, United States of America

## Abstract

Trudo Lemmens and colleagues discuss how the mere suggestion of litigation can bias the medical literature by affecting editorial decisions. They argue that journals and authors should publicly post threats of litigation, or cease and desist letters, for which there are some international legal precedents.

*Please see later in the article for the Editors' Summary*

**Summary Points**

- The mere suggestion of litigation can bias the medical literature by affecting editorial decisions.
- Journals and authors should publicly post threats of litigation or cease and desist letters.
- There are some international legal precedents for publicly posting litigation threats.
- Posting of litigation threats has some advantages over commonly employed strategies for guarding against libel chill, such as legal consultations and litigation insurance.

## Introduction

Editorial independence is critical if medical journals will continue to be venues for important debates that affect patient care. Editorial independence includes the latitude to accept or reject manuscripts on the basis of their content and without fear of repercussions against the journal. That independence is diminished when concerns about libel lawsuits deter journals from publishing contentious manuscripts. The Supreme Court of Canada has described this phenomenon of “libel chill” as follows: “There is concern that matters of public interest go unreported because publishers fear the ballooning cost and disruption of defending a defamation action. … When controversies erupt, statements of claim often follow as night follows day, not only in serious claims … but in actions launched simply for the purpose of intimidation. Of course ‘chilling’ false and defamatory speech is not a bad thing in itself, but chilling debate on matters of legitimate public interest raises issues of inappropriate censorship and self-censorship” [1]. Here, we explain why this “libel chill” effect is currently insufficiently countered with legal consultations and litigation insurance. We suggest that in the absence of significant reform of libel law, which is beyond the scope of this paper, medical journals post threats of litigation; and we discuss some legal implications of doing so.

## When Can Threats of Libel Affect the Publication Process

It is difficult to determine how often concerns about litigation prevent manuscripts from being published or impact significantly on the content of manuscripts. In 1995, a Report of the Conference to Promote International Cooperation among Medical Journal Editors opined that “[e]ditorial freedom is, first and foremost, a freedom from a number of threats,” and that “[t]hreat of lawsuits against journals and editors seems to be an increasingly relevant and chilling factor in some countries” [2]. Since then, little has been done to counter libel threat, and there are indications it continues to be a serious concern, perhaps even more so in the context of the multiple controversies surrounding the safety and efficacy of health care products, which are often aggressively promoted by companies that have the financial means to start legal action. The journal *Science*'s informal 2010 survey of 22 leading scientific and medical journals found that several journals “reported rejecting papers that were ‘clearly libelous’ or removing material that might have attracted libel action,” though “they insisted that this had been done on editorial grounds” [3]. American Psychological Association publisher Gary VandenBos estimated that he dealt with “about 20 to 30 threats of lawsuits related to manuscripts in prepublication status” in 25 years [3]. In 2001, the editor of the *New Zealand Medical Journal* left a blank space in an article with the note: “the paragraph was withdrawn for legal reasons” [4]. Two of the authors of this article have been confronted with either editorial decisions not to publish an article on a controversial topic or with removal of sections of an article just prior to publication because of the editors' fear for legal liability.

It is plausible that parties with large sums of money to gain or lose based on the content of a medical journal article (e.g., pharmaceutical or device companies) may attempt to exert their influence over editorial decisions with cease and desist letters or threats of litigation. This behavior can have a significant impact on editorial decisions, because of the power imbalance between a well-resourced and keenly motivated adversary and an often under-resourced journal with more submissions than can be published. Smaller journals may not be able to survive even the mere litigation process because of legal defense costs, as one of us was told by an editor when inquiring about the last minute pre-publication removal of a paragraph from an article published in the journal. While threats of lawsuits or subpoenas have been used to intimidate authors and even peer reviewers [5],[6], editors and publishers may also be put under pressure; they may be reluctant to publish controversial articles or editorials because of threats or fear of lawsuits even when authors are willing to take the risk.

## Current Responses

A natural first response to a legal threat is to consult a lawyer. Journal editors are not generally professionally trained to deal with libel threats and even those with formal training may not have experience dealing with such threats. Obtaining such advice can improve the quality of a publication. As one scientific journal publisher quoted in *Science* reported: “We know that on occasion academics may make assertions they can't then support; the legal advice we obtain often helps them to clarify their thinking and so we end up with a better paper, as well as one that should stand up in any court” [3].

Insurance may help journals defray the high cost of such legal advice. There are, however, two limitations to insurance schemes. The first is that insurance costs generally scale with the insured's perceived risk profile—in this case, its history and likelihood of attracting legal challenges. Journals publishing in controversial areas or willing to take a stance may be more likely to incur higher insurance costs. The second related problem is that insurance companies often exercise control over what approach to take when faced with legal action. They may insist on compromise or settlement, even when editors wish to press ahead with publication.

Even if legal advice is affordable it may not be helpful to curb the impact of libel threats. While there are differences in how lawyers approach these threats, one reasonable approach for lawyers consulted by journals about manuscripts that can attract litigation is to err on the side of caution. They tend to have a professional interest in avoiding future criticism that could arise if a journal is taken to court following their more lenient legal advice. Cautionary advice, resulting in rejection of the manuscript, is perhaps more likely when legal consultation is mandated by a libel insurance policy. Decisions about publication may thus be affected by opinions from lawyers who are unable to comment on the scientific merit of the manuscript and who think foremost about the insurance company's interest in avoiding a lawsuit.

## A New Proposal to Expose Libel Threats

We recommend that journal editors consider the option of publicly posting litigation threats, e.g., cease and desist letters or more subtle threats that are received either when a manuscript is being considered for publication or after it has been published.

The aim of this practice is deterrence and accountability rather than retaliation. Posting cease and desist or litigation threat letters will empower journals to publish worthy manuscripts despite threats and discourage specious claims meant to intimidate journals. The registry will expose such threats to scrutiny by the public, the government, and the press. Note that people or organizations will still be able to protect themselves against unwarranted allegations, as posting threats of litigation should not deter parties with legitimate concerns about being defamed from pursuing legal action. These should be able to publicly explain without embarrassment why they are pursuing legal action. In contrast, companies have an interest in avoiding exposure of their aggressive legal tactics if there is a weak basis for a threat. Making these threats transparent also promotes accountability of journal editors. A journal that refuses to publish an article after receiving a cease and desist or litigation threat letter can be pushed to explain its rationale. Posting threats promotes open discussion about freedom of speech by bringing to light a troubling aspect of research. It would, in our view, be a way to live up to what the Committee on Publication Ethics (COPE), an advisory body with a membership of over 8,000 journals, recognizes to be among the “[g]eneral responsibilities and duties of editors” to “*champion* freedom of expression” [7].

This action can, of course, only work if many journals actually facilitate publication of these letters. The deterrent effect of the practice depends on its widespread adoption such that parties contemplating intimidation tactics actively take it into consideration. Organizations such as the International Committee on Medical Journal Editors (ICMJE), COPE, or the World Association of Medical Editors (WAME) could support this approach and promote adherence by making it a condition of membership. These organizations could also offer certifications to non-affiliated journals for their commitment to this approach, while these journals could also explicitly identify it as an essential component of their editorial policy. Furthermore, these organizations could take the lead in establishing and maintaining, with support of their members, a special website dedicated to this form of exposure of libel threats directed against medical journals. The advantage of the jointly organized website would be the ease of access for posting and for analyzing libel threats, the increased visibility of the initiative, and the sharing of maintenance costs and costs of initial legal consultations.

Threat letters can be posted without comment on the merits of the threat and—where needed—with identifying information redacted. Editors should discuss threat letters and carefully assess how to publicize the letters in a way that squares with editorial integrity and risk management. Redaction of threatening letters to remove identifying information about the authors and the type of publication that is the target of a threat could be necessary if the decision is made not to publish a paper. The reason is that publicizing details about the rejected paper could undermine the anonymous submission and evaluation process of journals and could compromise the submission of the paper to another journal. In that case, an editorial note could accompany the publication of the letter, explaining in as much detail as possible the type of threat and the nature of the publication. An editorial note could also identify why the journal ultimately rejected the paper, whether it is because of the threat or for other editorial or quality assessment reasons. Of course, journals should consult legal counsel both before adopting any publication policy, as well as with respect to specific letters and threats.

The specifics of a registry of threatening letters requires further elaboration with input of the editorial boards of leading medical journals. But it is worth pointing out here that there are precedents for this approach. One existing registry with a comparable objective, but focusing on internet postings and online publications—not on pharmaceutical and medical publications—is ChillingEffects.org, a website maintained by a coalition of American law school clinics and the Electronic Frontier Foundation [8]. The site archives letters and notices from a number of categories; as of May 9, 2013, there were 104 notices or letters categorized as involving alleged defamation [9]. Google also publishes the copyright infringement “takedown” notices it receives at ChillingEffects.org [8]. The founders of ChillingEffects.org describe the important role their registry has played as a source of impartial data to inform public understanding of the copyright takedown process, as well as scholarly and policy debate concerning the same [10].

## Possible Risks of Publishing Libel Threats

Editors or those in charge of organizations that would take on the task of developing websites posting libel threats should carefully explore with expert counsel the legal consequences of publishing libel threat letters in all relevant jurisdictions, not just their own. Libel laws and other sources of liability have long arms such that what is safe under the laws of one jurisdiction may fall within the extraterritorial reach of another. We realize that this means that initially only journals or organizations with significant financial resources may feel comfortable to take the lead with this approach. Yet, there are promising indications and precedents in some Common Law jurisdictions that suggest it is a feasible approach that should not expose them to significant financial risks. We briefly discuss here selected examples from Canada and the United States as a discussion of implications in all countries is beyond the scope of this article.

In Canada, a 2006 case from Alberta, *Angle v. LaPierre* [11], suggests that merely republishing a cease and desist letter that contains allegations of defamation *without comment on the letter itself or the underlying issue* is not by itself defamatory.

The *Angle* case dealt with a lengthy series of disputes between individual teachers and a teachers' association. The latter was suing the teachers for public statements concerning the disputes made on a number of public fora, including a website. The teachers had, among other things, republished a letter by the teachers' association in which it threatened legal action if they did not stop voicing their criticism in public, accompanied by the teachers' responses to those letters. The association challenged in particular public statements that referred to its tactics as legal or institutional bullying aimed at curtailing freedom of speech.

While the court recognized that these accusations were “defamatory,” it ruled that several of them were protected as “fair comment,” since the legal tactic of the cease and desist letter could fairly be qualified as an attempt to “coerce a change in behaviour” [11]. “To the extent there is a defamatory sting in this publication [of the cease and desist letter and commentary]” the court ruled, “it is opinion based upon fact on the general subject of education and the role of the [teachers' association] in that system [which] are matters of public interest” [11]. Interestingly, the court emphasized in its assessment of the fairness of one of the statements the fact that it did not elaborate on the merits of the dispute occasioning the tactic, but was merely focusing on the legal tactic itself. According to the court, “*the conversation that ensued … was fair comment on the contents of the cease and desist letters*. None of it was comment on any of the specific instances and events that prompted issuance of those letters” [our emphasis] [11]. By contrast, the court found that other comments suggesting that parents would feel threatened “for asking questions about the education of their children” were misrepresenting the possible consequences of the cease and desist letters and were thus defamatory [11].

The court found that a forceful opinion stating that plaintiffs' letters “threatening legal action” constituted an attempt to silence opponents was protected as fair comment in part because it did not delve into the content of the controversy. By that same logic, republishing such letters without *any* comment, and with private information redacted, would have been even more defensible. Of course, *Angle* also serves as a cautionary tale, for it makes quite clear that exchanges that ensue from the publication of letters could attract liability. Moreover, the *Angle* case is a lower court decision, and thus not binding on other courts. Yet it shows how the law of defamation has been applied favourably by at least one Common Law court to shield the publication of litigation threats.

In the Unites States, which for several decades has had what some view as “the most speech protective substantive libel laws in the world,” [12] a recent legal exchange between Public Citizen Litigation Group—the consumer organization founded by Ralph Nader—and the company DirectBuy reflects how consumer organizations have confidently resisted efforts to block republication of litigation threats by alleging copyright in the threat letter. DirectBuy had sent letters to the operator of several consumer complaint sites, demanding that the operator remove and cease making allegedly defamatory claims. The letter concluded with a warning that the letter itself was copyrighted by the firm—the letter had been registered with the Copyright and Trademark Office—and that unauthorized republication would result in additional legal claims.

Media law scholar Sam Bayard at Harvard University's Berkman Center for Internet and Society concludes his discussion of the issue by stating: “Don't be bullied by a lawyer threatening you with a copyright infringement suit [under American law] for republishing the contents of a threatening letter. One way or another, this is an extremely weak legal argument, and one that the lawyer is extraordinarily unlikely to pursue” [13]. ChillingEffects.org similarly notes, “[i]t is highly unlikely that someone could sue successfully [under American copyright law] for the posting of a cease-and-desist notice (most notices are minimally creative; the use is for purposes of commentary and research; the amount used is necessary to the understanding; and there is no effect on a ‘market’ for cease-and-desist letters)” [14].

There is also the possibility that attempts to sue those who post litigation threats would actually result in greater public scrutiny of the larger issue of “libel chill” and could result in changes to the law, just as high profile defamation cases in the United Kingdom led to changes in the law that now protect important scientific debate [15]. Moreover, the law and those applying it must recognize that public policy militates strongly against liability for republishing litigation threats. Years of media coverage of the issue make it clear that the entire phenomenon of libel chill is eminently a public interest issue; debate about that topic is impoverished if people cannot reveal specific instances of it.

## Conclusion

Medical journals should explore the option of bolstering their commitment to editorial independence by publicly posting threats of litigation. Consultations with lawyers and litigation insurance may reduce the likelihood of lawsuits but neither insulates editorial decisions from libel chill. While exposing litigation threats will not completely prevent them, it may be a step towards exposing the problem, increasing accountability, and fostering new social, scholarly, and legal norms.
